# The Day-Hospital of the University Hospital, Bobo Dioulasso: An Example of Optimized HIV Management in Southern Burkina Faso

**DOI:** 10.1371/journal.pone.0125588

**Published:** 2015-05-13

**Authors:** Julie Chas, Arsène Hema, Laurence Slama, Nongondo Firmin Kabore, François-Xavier Lescure, Camille Fontaine, Gilles Pialoux, Adrien Sawadogo

**Affiliations:** 1 Service des Maladies Infectieuses et Tropicales, Hôpital Tenon, Paris, France; 2 Hôpital de Jour, CHU Sourô Sanou, Bobo Dioulasso, Burkina Faso; 3 AP-HP, Service de Maladies Infectieuses et Tropicales et IAME, Hôpital Bichat-Claude Bernard, UMR 1137, INSERM, Univ Paris Diderot, Sorbonne Paris Cité, F-75018 Paris, France; 4 Centre 190, Paris, France; 5 UPMC, Paris, France; Pasteur Institute in Cambodia, CAMBODIA

## Abstract

**Objectives:**

To evaluate the epidemiological evolution of patients with HIV (PtHIV), between 2002 and 2012, in a day-hospital that became an HIV reference centre for south-west Burkina Faso.

**Materials and Methods:**

This was a retrospective study of PtHIV followed in the Bobo Dioulasso university hospital since 2002. The study was based on clinical data recorded using ESOPE software and analysed using Excel and SAS.

**Results:**

A total of 7320 patients have been treated at the centre since 2002; the active file of patients increased from 147 in 2002 to 3684 patients in 2012. Mean age was stable at 38.4 years and the majority were female (71%). The delay to initiation of antiretroviral (ARV) treatment after HIV diagnosis decreased from 12.9 months in 2002 to 7.2 months in 2012. The percentage of PtHIV lost to follow-up, untreated for HIV and deaths all decreased after 2005. Voluntary anonymous screening and/or an evocative clinical picture were the main reasons for HIV diagnosis, usually at a late stage (41.1% at WHO stage 3). Virological success increased due to a decrease in time to initiation of ARV treatment and an increase in percentage of patients treated (90.5% in 2012, mainly with 1^st^ line drugs). However, there was also a slight increase in the rate of therapeutic failures and the percentage of patients who progressed to 2^nd^ or 3^rd^ line-ARVs.

**Conclusion:**

Our day-hospital is a good example of the implementation of a specialist centre for the management of PtHIV in a resource-limited country (Burkina Faso).

## Introduction

In 2012, the UNAIDS report estimated that 35.3 million individuals worldwide were living with the human immunodeficiency virus (PtHIV), representing a prevalence of 0.8% [1]. This figure has increased constantly since 2001, particularly in sub-Saharan Africa.

Burkina Faso is one of the countries in sub-Saharan Africa that has the lowest prevalence of HIV. The number of PtVIH was 120 000 [IQR: 100 000–150 000] in 2013, representing a prevalence of 1% in the general adult population (15–49 years) [2]. In 2003, this prevalence was 1.8%. The general trend shows a stabilization of the epidemic since 2005, with a prevalence of 1.2% in women and 0.8% in men of the same age group and 2.1% in urban areas versus 0.6% in rural areas. Cover with antiretroviral (ARV) treatment (ART) is 79%, corresponding to an increase of 46% since 2006 [3]. Despite the massive scale-up of combination ART (cART) in low-income and lower middle-income countries, CD4 cell counts at cART initiation have increased slightly [4], particularly after 2010 when the WHO Guidelines for resource-limited settings were revised, increasing the CD4 cell count threshold for cART initiation in asymptomatic HIV-positive patients from 200 to 350 cells/mm^3^ irrespective of clinical symptoms [5–6]. A study of the International Epidemiological Database to Evaluate AIDS in Southern Africa (IeDEA-SA) has demonstrated that a lower CD4 count at cART initiation is associate with increased mortality [7]. Another study demonstrated that late ART initiation was significantly associated with mortality [8]. In addition, a medico-economic study showed that a higher CD4 cell count at cART initiation had a beneficial economic impact in rural Uganda [9]. An analysis of PEPFAR-supported HIV care clinics in eight sub-Saharan African countries found that CD4 cell counts at cART initiation increased as HIV testing coverage in the region increased [10]. The favourable evolution of these indicators in the fight against HIV is due mainly to a multi-sectorial and decentralized approach through an increase in number of centres providing ART for PtHIV. In 2012, there were 95 public health institutions, private or community, offering HIV management in the 13 regions of the country [2].

Since 1997, the university hospital (CHU) of Bobo Dioulasso, the second city in Burkina Faso, has treated PtHIV. In 2005, a day-hospital was created, in partnership with the authorities in Paris and supported by the program “Ensemble pour une Solidarité Thérapeutique Hospitalière En Réseau” (ESTHER) in a North/South partnership with the Infectious Diseases Service of the CHU, Tenon (AP-HP, Paris, France). The day-hospital of Bobo Dioulasso specializes in the multidisciplinary management of PtHIV and was the object of decentralization for the southern region of Bobo Dioulasso.

The aim of this study was to describe the 10-year epidemiological trends in access to HIV-care between 2002 and 2012 (ARV coverage, delay between HIV diagnosis and CD4 count at the start of ART) and ART outcomes (WHO progression, immunological success, virological success) among HIV-infected adults followed in an HIV day care hospital in Bobo-Dioulasso, Burkina Faso.

## Methods

### Study design

This was a retrospective, observational study carried out on a cohort of PtHIV followed in the Internal Medicine Department of the CHU, Bobo Dioulasso, from 1997 to 2005, and then followed in a day-hospital in Bobo Dioulasso from January 2005 to December 2012. The patients included and analysed in this study were those who were in care at any time during the two study periods.

The university hospital Souro Sanou (CHUSS) of Bobo-Dioulasso is the reference centre for the Western region of Burkina Faso. From 1997 to 2005, two medical consultation rooms in the Internal Medicine Department of this hospital were dedicated to the management of HIV-infected adult patients, representing a state of « medico-social permanency ».

On 25 July 2005, the activities of these two rooms were transferred to the current day-hospital of Bobo-Dioulasso. The day-hospital is a decentralized operational department of the CHUSS. It was formed on the initiative of the Fondation Jacqueline BEYTOUT and the Organisation Panafricaine de Lutte contre le SIDA (OPALS). In this centre, PtHIV receive free management of HIV: medical consultations, therapeutic education, biological examinations, dispensing of ARVs and medicines against opportunistic infections.

The day-hospital of Bobo-Dioulasso, like the previous consultation rooms, receives HIV-positive adults who are referred from diagnostic centres in the town of Bobo-Dioulasso and from hospital departments in the CHU, Bobo-Dioulasso. All patients arriving at the day-hospital are received by a mediator who then introduces the patient to the physician for a medical consultation. The patient undergoes a complete clinical examination and paraclinical examinations are prescribed when necessary. The decision to start treatment with ARVs complies with WHO recommendations.

The day-hospital laboratory is equipped with a biochemistry analyser (KONELAB), a serology-immunology analyser (AXSYM), a haematology machine (BECKMAN COULTER), a CD4 counter (FASCOUNT) and two real-time PCR machines for measuring the plasma HIV viral load by two techniques (Biocentric with a threshold of 300 copies/μl and Abbott with a threshold of 40 copies/μl).

The therapeutic education consultation started in 2010, after the training of its participants (nurses and mediators). Each patient received at least two therapeutic education consultations before the start of their ARV treatment. Compliance was measured by counting the number of pills brought back to the centre every 3 months. The pharmacist or the pharmacist’s assistant is responsible for dispensing the ARVs and the medicines for opportunistic infections. The patient presenting at the pharmacy is registered in the pharmacy database (LOGONE). LOGONE software, used by the pharmacy, enables the real-time location of patients at risk of stopping treatment and the active search for patients lost to follow-up. The latter is also assured by PtHIVs who serve as an interface between patients in the community and the HIV management team. These PtHIVs are trained in mediation in health.

### Study population

The study population consisted of all patients in the medico-social permanency set-up and then in the day-hospital who were registered in the Bobo-Dioulasso day-hospital database.

### Data collection

No ethics committee approval was required for this retrospective study, but the study was approved by the institutional board of the day-hospital of the CHUSS. Patient records/data were anonymized and de-identified prior to exporting and analysis using an encoding process (6-digit number without specifying the date of birth). There were two evolutionary phases in the method of collection of routine data in the day-hospital of Bobo-Dioulasso. From 1997 to 2006, data relating to medical visits were collected in exercise books serving as medical files. This method of data collection was neither standardized nor exhaustive. In addition, the large number of patients followed made evaluation of the management of PtHIV arduous. To respond to the demands of follow-up evaluation, computer management of medical data was introduced in 2006. The data in the medical files were collected retrospectively using ESOPE software (Evaluation et Suivi Opérationnel des Programmes Esther, Epiconcept, France) by data entry operators. The exercise books were replaced by standard medical files inspired by the ESOPE software interface to facilitate the coding of different data. From 2007, real-time storage of medical data on ESOPE was assured by the physicians.

This software is currently used in 11 African countries and 17 centres in Burkina Faso. The large number of missing data before 2002 justified the start of this retrospective analysis from this date onwards. In 2007, LOGONE (Entrepreneurs du Monde) software was also installed in the pharmacy which manages the dispensing of ARVs. This enabled the follow-up of patients taking ARVs and helped to identify those lost to follow-up or those who had discontinued treatment.

Patients no longer receiving ART made at least two medical visits per year. Patients receiving ART were seen 2 weeks after the start of treatment, at 1 month, 3 months, 6 months and then every 6 months for patients who tolerated their treatment well.

CD4 counts were measured at the first visit and then every 6 months in patients not receiving ARVs. In those receiving ARVs, CD4 counts are measured at 3 months and 6 months of treatment and then every 6 months. Measurement of viral load started in 2008 and took place every 6 months in patients on ARVs. Each consultation report was compiled in the patient’s file (before 2007) or produced in real-time by the physician using ESOPE (since 2007).

### Operational definitions

Patients were declared lost to follow-up if they were absent from the weekly follow-up appointment and from renewal of ARVs, and were not found after 6 months search carried out by health mediators.

A plasma HIV load of <300 copies/ml was considered to be undetectable.

Untreated patients with HIV, enrolled in the HIV care programme and not started on treatment (pre-ART patients) were identified.

### Statistical analysis

The data analysed were obtained from medical consultations, the on-site laboratory (for biological specimens) and the pharmacy dispensing ARVs and treatments for opportunistic infections.

The data collected on ESOPE were exported to Excel and STATA 12 for statistical analysis. The large number of missing data before 2002 justified the start of the retrospective analysis from this date. Socio-demographic, clinical, biological and follow-up data for the population were described according to the year of recruitment. Qualitative variables are described as percentages and quantitative variables as median values and interquartile range (IQR).

Fischer's exact test and the Mann Whitney test were respectively used to compare qualitative and quantitative variables of two independent groups.

The Chi2 test for trend was used to compare categorical variables between years and the trend Cuzick test to compare continuous variables between years. Survival of patients in cohort was investigated by considering the loss to such deaths and more cases reported deaths. We used the Kaplan-Meier method to describe the survival in the cohort HDJ 2002 to 2012. The survival curves of the different annual cohorts were compared by the test Breslow-Gehan-Wilcoxon.

## Results

### Socio-demographic data

**Fig 1:**
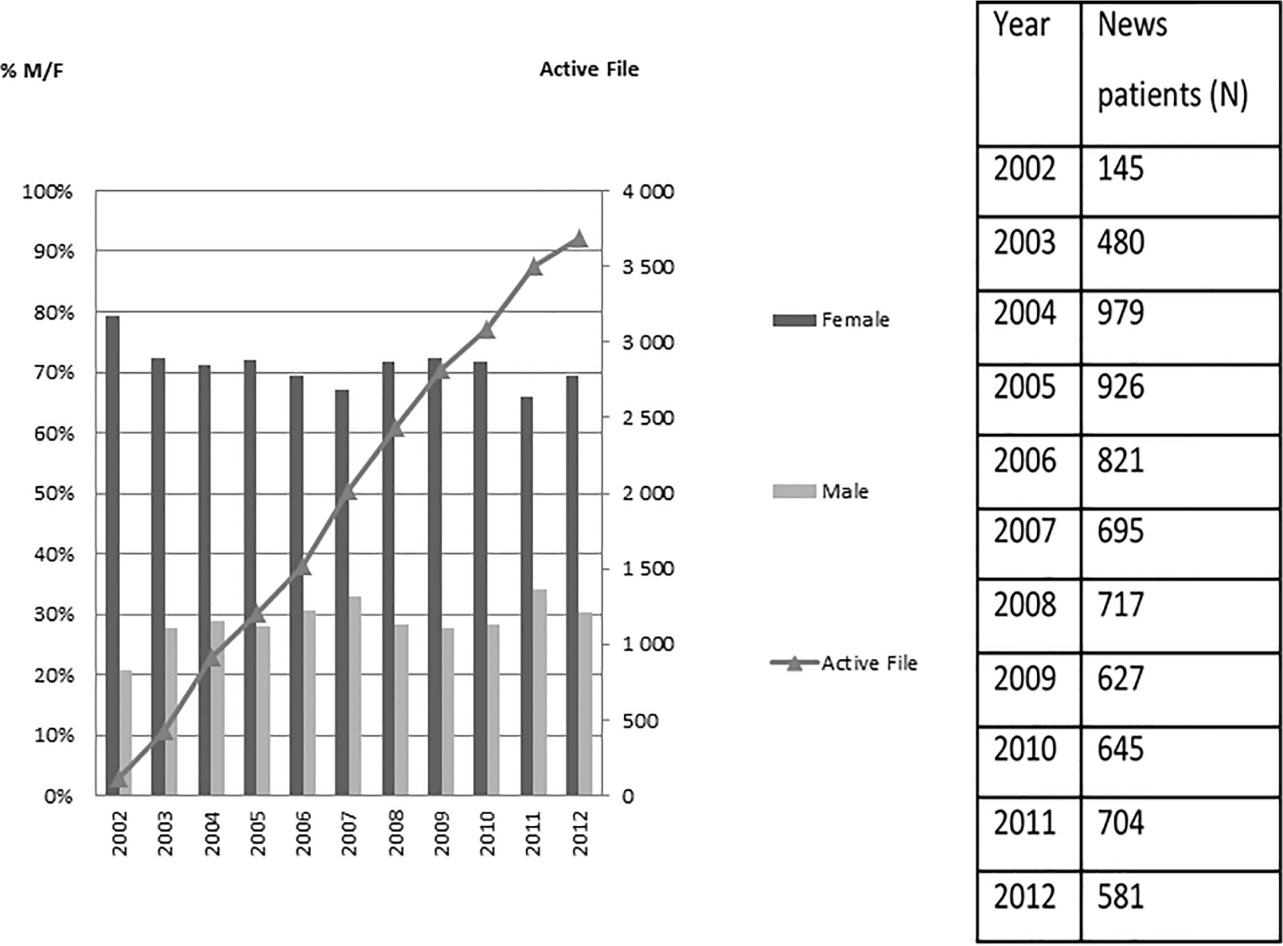
Socio-demographic characteristics of the study population.

Over the 10 years of the study, the active file of PtHIV increased constantly from 147 patients in 2002 to 3684 patients in 2012. The median number of new cases per year was 695 [IQR: 145–979] and the number of news patients diagnosed yearly is reported in the Fig 1. The male/female ratio remained stable with a mean of 71.1% women and 28.9% men (Fig 1). Similarly, age also remained stable with a median age of 38.4 years [IQR: 31–44 years]; however, there was a trend towards older age in men (43 years; IQR: 37–48 years) than in women [36.6 years; IQR: 30–42 years] p<10^–4^. Seropositivity to HIV mainly concerned patients aged 15-45-years (78.2%) (only 21.8% of those >45 years).

In terms of education, 47.6% of patients were illiterate, 26.9% had attended primary school, 23.7% had attended secondary school and only 1.8% had received further education.

In terms of marital status, 20.8% patients were single, 1.8% were living with a partner, 42.1% were in a monogamous marriage, 7.5% were in a polygamous marriage, 21.2% were widowed and 6.6% were divorced. There was an average of 2.2 children/female.

There was no significant change in the socio-demographic data over time.

### HIV follow-up

**Fig 2:**
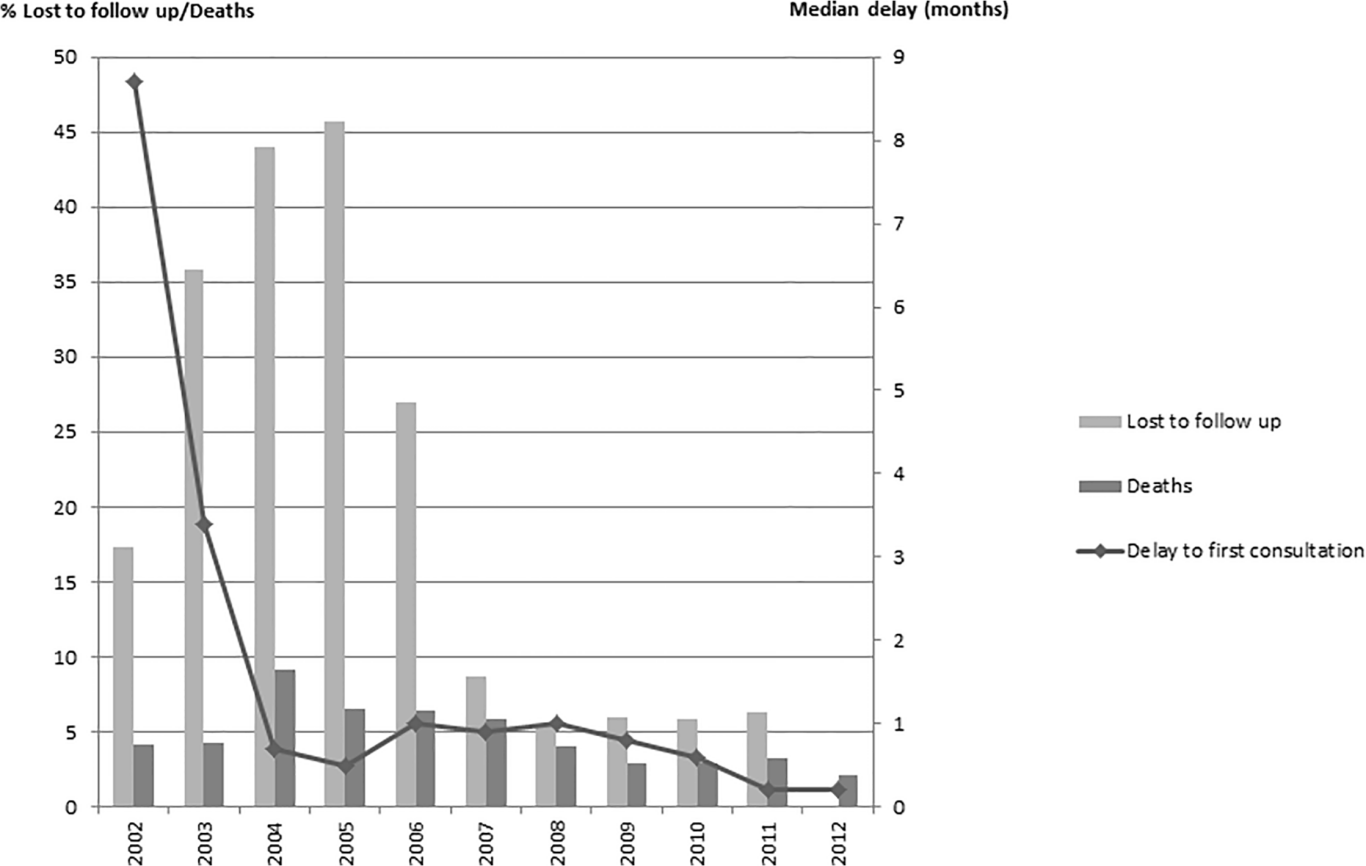
Data for follow-up of HIV.

The median time between HIV detection and the first consultation decreased from 8.7 months [IQR: 4.4–14.4 months] in 2002 to 0.2 months [IQR: 0.1–0.7 months] (p<10^–4^)in 2012 (Fig 2).

The percentage of patients lost to follow-up increased from 2002 to 2005 (45.7%) and then decreased constantly to 6.3% in 2012 (Fig 2). The majority of these patients were not receiving treatment with ARVs (72.9%).

**Fig 3:**
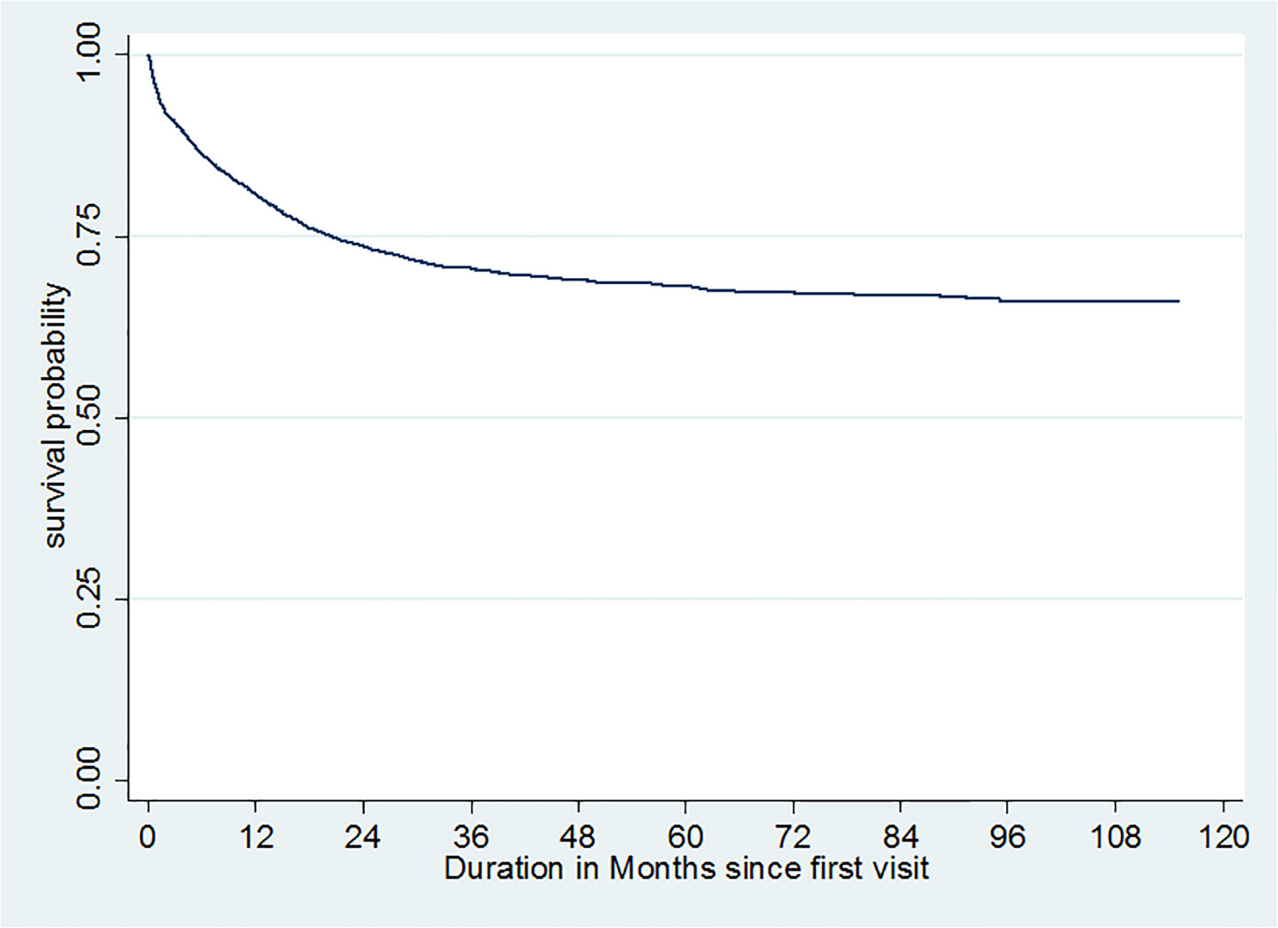
Survival probability curve among the HIV infected patients cohort at the Bobo-Dioulasso day hospital between 2002 and 2012.

**Fig 4:**
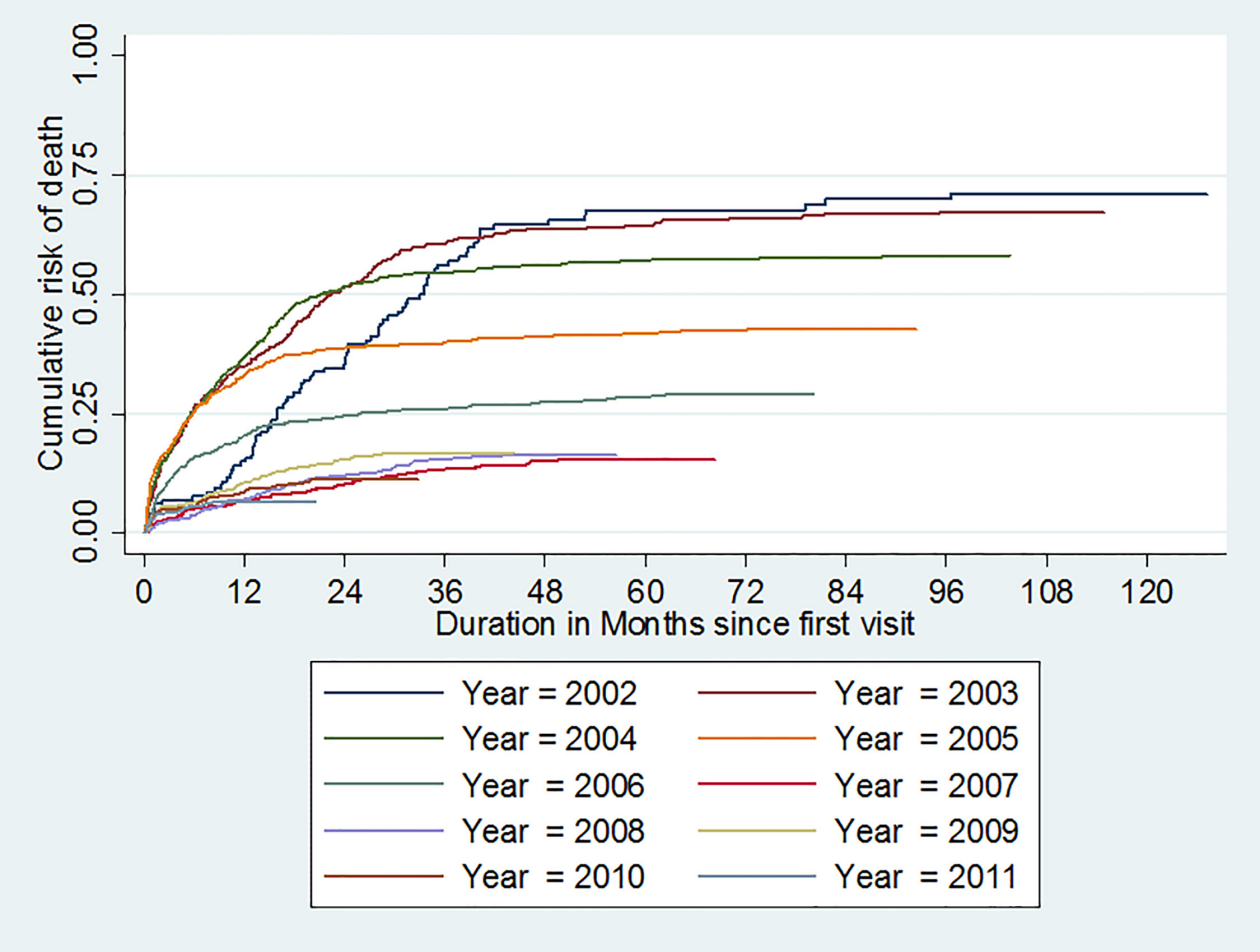
Survival probability curve according to antiretroviral therapy among the HIV infected patients cohort at the Bobo-Dioulasso day hospital between 2002 and 2012. (test de Breslow-Gehan-Wilcoxon P<10^–4^).

The annual mortality rate evolved in the same way between 2002 and 2012, with an increase until 2004 (9.1%) and then a decrease to 2.1% in 2012 (Fig 3 and Fig 4).

### Clinico-biological data at HIV diagnosis

**Fig 5:**
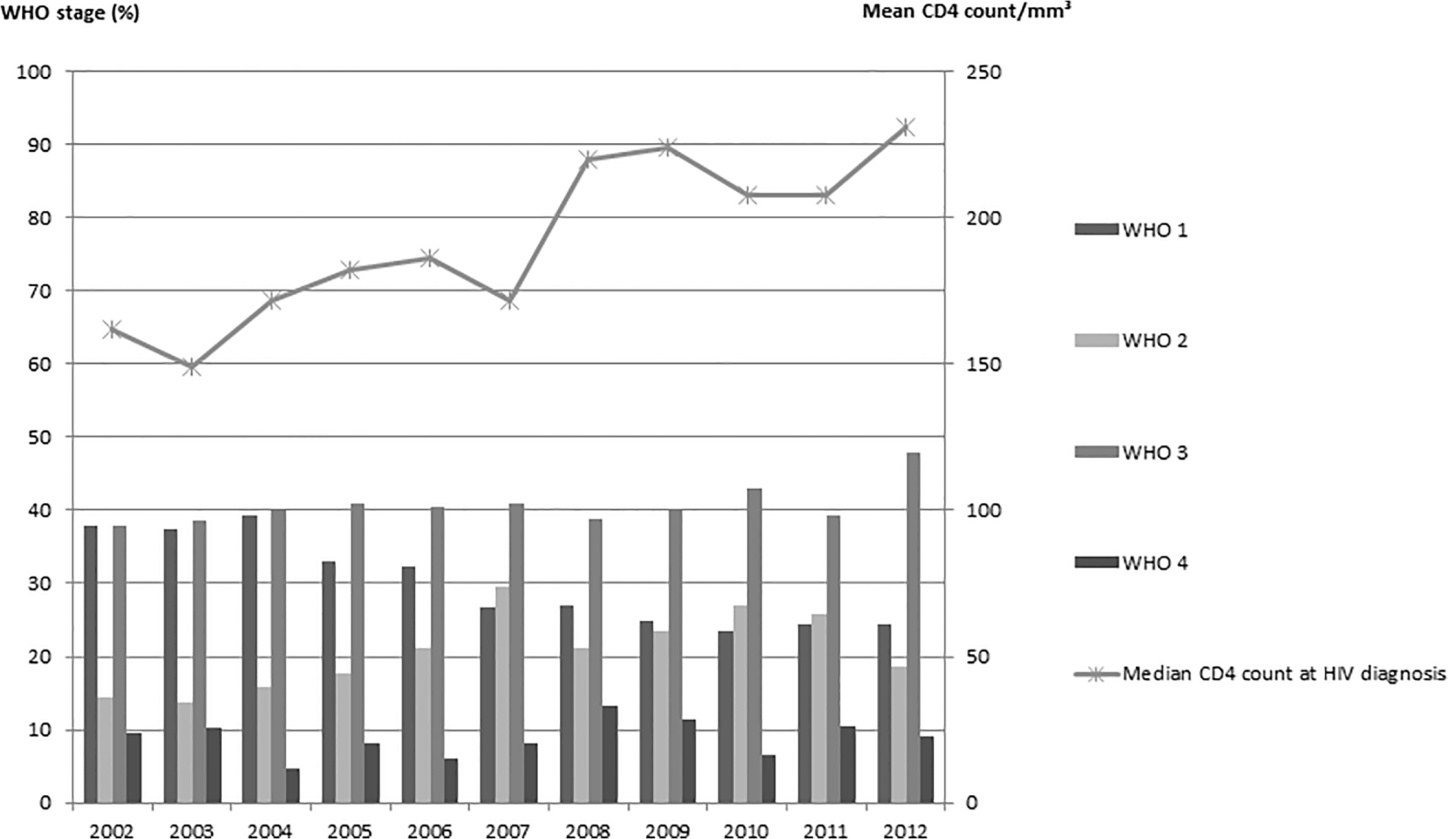
Clinico-biological data at HIV diagnosis.

In the day-hospital of Bobo Dioulasso, there were three main contexts for the detection of HIV seropositivity: voluntary and anonymous screening (52.7%), a suggestive clinical picture (44.4%) and the prevention of mother-child transmission (3.0%), with a similar distribution over time. Nevertheless, diagnosis remained late, with a similar distribution of the different WHO stages each year (Fig 5). In 2012, 48% of diagnoses were made at WHO stage 3, 24.3% at stage 1, 18.6% at stage 2 and 9% at stage 4.

The majority of patients were infected with HIV-1 (93.4%), 2.6% with HIV-2 and 4.0% were coinfected with HIV-1 and HIV-2.

Mean CD4 count at HIV diagnosis since 2002 was 241.8/mm^3^ (196.9/mm^3^ in 2002 and 267.7/mm^3^ in 2012 (p<10^–4^) (Fig 5).

### Data for treated patients

**Fig 6:**
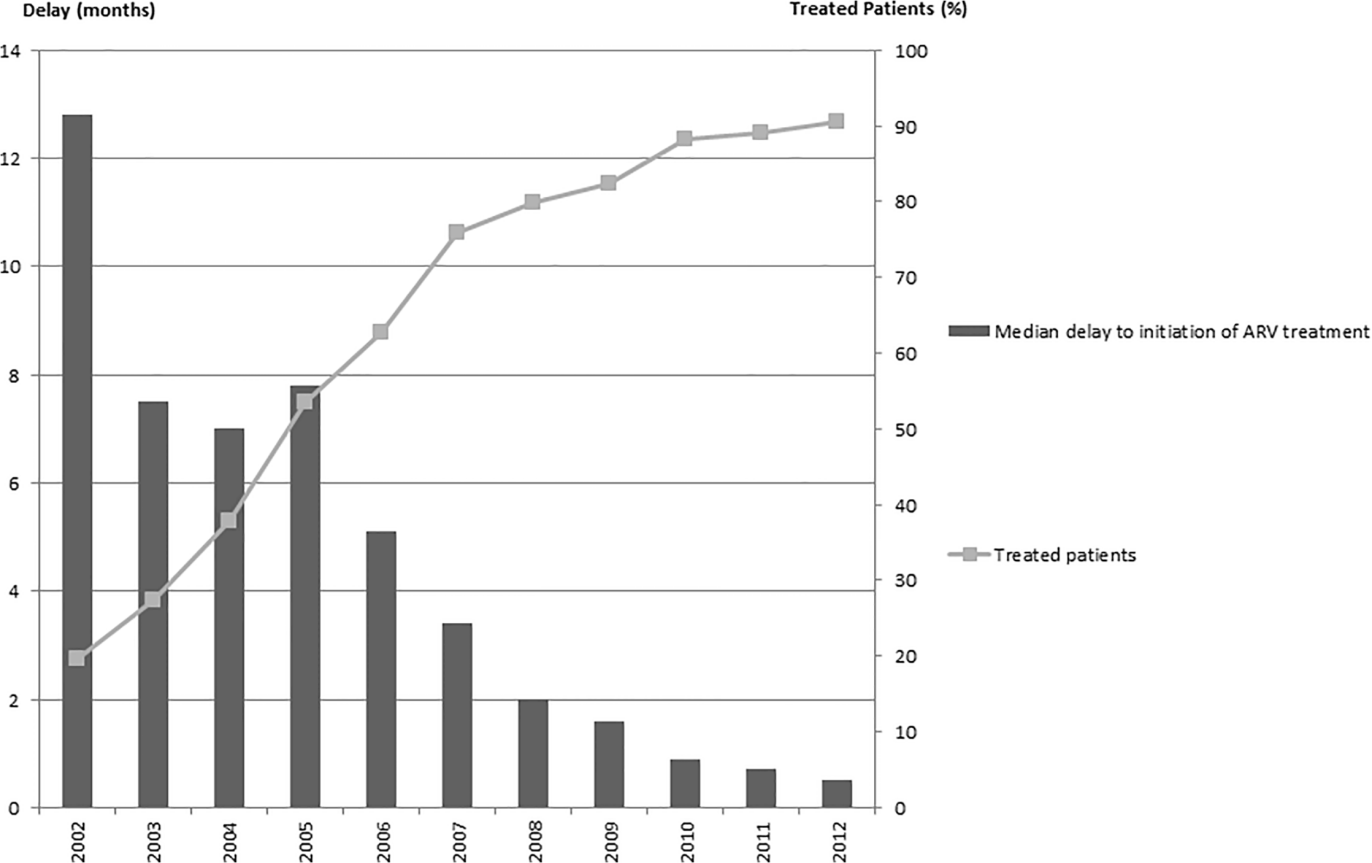
Data for patients started on antiretroviral (ARV) treatment.

Therapeutic cover with ARVs increased significantly from 121 patients (19.7%) in 2002 to 3335 patients (90.5%) in 2012 (Fig 6).

The median delay to initiation of ARV treatment from diagnosis decreased from 12.8 months [IQR: 6.0–19.5 months] in 2002 to 0.6 months [IQR: 0.3–0.9 months] in 2012 (p = 10^–4^) (Fig 4). This decrease in time to initiation of ARV treatment is characterized by a much higher median CD4 count at initiation of ARV treatment. This analysis was possible after 2005. Median CD4 count at the start of ARV treatment was 182/mm^3^ [IQR: 86-328/ mm^3^] in 2005 and >20% of patients had CD4 <50/mm^3^, whereas in 2012, median CD4 count was 230/mm^3^ [IQR: 106-371/ mm^3^] (p = 10^–4^) and 16% of patients had CD4 <50/mm^3^ [4].

Over three-quarters (84%) of treated patients had virological success (viral load <300 cp/ml) and 16.0% had virological failure, with 2.5% having a viral load between 300 and 1000 cp/ml, 2.7% between 1000 and 10 000 cp/ml, 4.8% between 10 000 and 100 000 and 6% >100 000 cp/ml.

In 2012, most of the treated patients were receiving first-line ARV treatment (94.9%), 5.1% were receiving second-line and 0.04% third-line treatment.

The most widely used combination of ARVs used in 2012 was the association of two nucleoside reverse transcriptase inhibitors (NRTIs) with a non-nucleoside reverse transcriptase inhibitor (NNRTI) in 80.4% of cases. In 19.5% of treated patients, the ARV combination was the association of two NRTIs with a norvir-boosted protease inhibitor. Finally, 0.08% received two NRTIs plus an integrase inhibitor.

The most widely used combination of NRTIs was 3TC + AZT (lamivudine/zidovudine) in 62.1% of cases. The most widely used NNRTI was efavirenz (EVF) in 54.5% of cases, followed by nevirapine (NVP) in 45.5% of cases. The most widely used protease inhibitor was lopinavir boosted by norvir (79.8%) and then indinavir (10.4%). Finally, the most widely used integrase inhibitor was raltegravir.

The distribution of the different therapeutic lines of ARVs used in 2012 had changed since 2002 with the availability of new molecules. The most widely prescribed ARV combinations were 3TC/AZT/EFV (26.3%) and 3TC/AZT/NVP (26.3%) (Table 1).

**Table 1:** Distribution of ARV treatment lines given to patients (up to 31/12/2012).

Therapeutic lines	Patients N(%)
AZT/3TC/EFV	867 (26)
AZT/3TC/NEV	867 (26)
FTC/TDF/EFV	300 (9)
AZT/3TC/LPV/RTV	300 (9)
3TC/D4T/NEV	167 (5)
3TC/TDF/EFV	134 (4)
3TC/D4T/EFV	100 (3)
Others	600 (18)

AZT: zidovudine; 3TC: lamivudine; EFV: efavirenz;

NEV: nevirapine; FTC: emtricitabine;

TDF: tenofovir disoproxil fumarate; LPV: lopinavir;

RTV: ritonavir; D4T: stavudine.

In terms of second-line treatment, the molecules used since 2006 were those recommended by WHO (i.e. ABC+DDI+LPV/r or TDF+FTC+LPV/r). Since 2010, the combination TDF+FTC+DRVr has also been available via the LADY 2 research protocol.

### Data for untreated patients

The proportion of untreated patients decreased between 2002 and 2012, from 80.9% in 2002, to 51.8% in 2005, 30% in 2007 and 9.5% in 2012.

Conversely, median CD4 counts in these patients at diagnosis increased from 104/mm^3^ in 2002 [IQR: 72-441/mm^3^] to 386/mm^3^ [IQR: 252-573/mm^3^] in 2005, 488/mm^3^ [IQR: 356–639] in 2007 and 544/mm^3^ [IQR: 458-594/mm^3^] in 2012 (p = 10^–4^) [4]. In parallel, the proportion of patients with CD4 <200/mm^3^ decreased from 51.6% in 2002 to 37.7% in 2005, 29.4% in 2007 and 16% in 2002 [11].

Before 2006, there was not enough treatment available to treat all eligible patients. However, from 2006 all patients responding to WHO criteria received ART if they wanted it.

## Discussion

The day-hospital in Bobo Dioulasso, with an active file of 3684 patients followed in 2012, represents a successful model for the management of PtHIV in sub-Saharan Africa. The latest socio-demographic data to be described in this part of Africa are similar to those observed in our study [12–13], with a male/female ratio of HIV patients in favour of women. This can, in part, be explained by the introduction of systematic screening for HIV in prenatal care centres. The steeper increase in coverage of HIV testing among women compared to men may be explained by the scale-up of programs designed to prevent mother to child transmission (PMTCT) [14]. In a systematic review and meta-analysis, men were more likely to be lost to the program and less likely to start cART than women [15]. This leads to the question about the best methods to develop to increase access to care for men infected with HIV [16], to obtain a better rate of « linkage of care » [17–18] and better compliance [19], as well as to discuss the circumstances for progression to second-line treatments in developing countries.

The management of PtHIV in this centre is characterized by a net decrease in time (between the date of HIV diagnosis and the first consultation) to the initiation of ARV treatment since 2002, which can be explained by an increase in number of medical and paramedical personnel in the Bobo Dioulasso centre, improved access to training on HIV management and a better structural organization for the reception of PtHIV. Late consultations with a view to obtaining ARV treatment remain an important factor in morbidity and mortality [20]. This is why the early detection of HIV is an important first stage in the early initiation of treatment and why fewer individuals should be “lost to follow-up” between diagnosis and the start of ARV therapy [15, 21].

According to our study, the number of patients lost to follow-up has decreased since 2005. In addition to a decrease in time to treatment of these PtHIV, this decrease in patients lost to follow-up can be explained by the use, since 2007, of software for the administration of ARV dispensing, which makes it easier to identify patients on ARV who do not present at the pharmacy to renew their treatment. The number of patients lost to follow-up in the day-hospital of Bobo Dioulasso is very low in comparison to the figure reported in countries in southern Africa in a recent literature review (60%) [22], these patients represent an important problem in the cohorts followed [22, 23]. A cohort study carried out in Southern Africa between 2004 and 2009 estimated the rate of follow-up between the first consultation and start of ARV treatment as 33% [24]. This demonstrates the importance of reinforcing systems of follow-up for these patients in decentralized centres.

Intensification of follow-up also enables the rapid initiation of ARV treatment, which can impact on the HIV epidemic in many regards, notably on mortality due to AIDS [25, 26]. In the specialist centre of Bobo Dioulasso, the number of deaths decreased due to the detection of HIV at an earlier WHO stage. This was due to an intensification of screening, strategic action plans in the fight against HIV/AIDS, closer monitoring of new patients and the rapid initiation of ARV treatment according to WHO recommendations. The intensification of ARV therapy can considerably modify the course of a national epidemic, by decreasing the viral load in the community, by stopping the proliferation of HIV and deaths linked to AIDS [27–29]. The therapeutic cover by ARVs in our day-hospital (90.5%) is greater than that observed in Africa (61%) [1] and more particularly in Burkina Faso as a whole (79%) [1, 2]. This expansion in ARV cover is a result of several factors: (i) ease of access, made possible by the introduction of free ARVs over the whole of the Burkina Faso territory since January 2010; (ii) the financial support of different partners including: the Global Fund to Fight AIDS, Tuberculosis and Malaria; UNAIDS; WHO; the President’s Emergency Plan for AIDS Relief (PEPFAR); as well as other partners in July 2013, in order to launch the initiative “Treatment of HIV” which aims to guarantee the success of objectives with regards to HIV treatment by 2015, with a view to achieving universal access to treatment [1]; (iii) to the decentralization of such a centre, making the services closer to the people [30]; and (iv) to the drawing up of clinical protocols allowing access to new molecules for patients in virological failure with the first-line treatments usually available. Abandoning the fees for biological analyses remains a preoccupation for community structures, particularly when 46.4% of the population lives below the poverty threshold, unemployment is around 77% and, with a gross national product per head in the order of 1 Euro/day/inhabitant, Burkina Faso remains a very poor country (ranked 181^st^ out of 187 in the latest classification of the human development index) [31].

Most of our patients received first-line treatment, but the rate of therapeutic failure is increasing in Africa with some patients already being treated with second- or even third-line ARV protocols [32, 33]. The evaluation of therapeutic efficacy and the demonstration of a possible failure depends on monitoring the viral load, which can be done on-site, but due to problems with the regular supply of reagents necessary for the frequent measurement of vial load, this evaluation is also based on monitoring the CD4 count and clinical monitoring. In the case of therapeutic failure, the association of two NRTIs with a norvir-boosted protease inhibitor is used as second-line treatment, and the combination of two NRTIs with an integrase inhibitor is used as third-line. The availability of molecules used for cases of therapeutic failure is made possible by clinical research studies in Africa, such as the clinical trials ANRS 12169 2-LADY and THILAO [34, 35]. All of these measures established by the day-hospital of Bobo Dioulasso follow the latest recommendations of WHO, published in June 2013 [36].

The majority of our treated patients were immuno-virological successes and were still receiving first-line treatment. This therapeutic success is explained by the basic but crucial therapeutic education of patients (TEP) introduced in our centre via multiple groups. According to data from 18 countries, the rate of compliance decreases over time: it is 86% and 72% at 12 and 60 months, respectively [37]. For this reason, TEP remains an important activity in this type of structure in sub-Saharan Africa and has an important impact of the evolution of the HIV epidemic in Africa [38, 39]. TEP is even more important in some groups of patients known to have a higher risk of poor compliance and therefore therapeutic failure [40, 41].

This study has several limitations and biases. First, because it is a non-comparative retrospective study, and secondly, because we used data that were collected routinely and missing date are inevitable. Thirdly, in including all patients who were in care at any time over the course of a given year to calculate LTF for that year (rather than calculating cumulative LTF for those newly enrolled by year of enrolment), we introduce a survivor bias. Finally no analysis could be conducted to estimate the effect of change in care over time or of the impact of specific aspects of care in improving patient outcomes

In conclusion, this study illustrates the excellent performance, over 10 years (2002–2012), of a hospital centre that has become a reference centre for the management of PtHIV in Bobo Dioulasso and a good example for sub-Saharan Africa. It demonstrates how to improve the reception, medical follow-up, psycho-social follow-up and quality of management of HIV and opportunistic infections in these patients.

## References

[1] Global report: UNAIDS report on the global AIDS epidemic 2013. Available: http://www.unaids.org/en/media/unaids/contentassets/documents/epidemiology/2013/gr2013/UNAIDS_Global_Report_2013_en.pdf.

[2] Global AIDS Response Progress Reporting 2012, Burkina Faso. Available: http://www.unaids.org/en/dataanalysis/knowyourresponse/countryprogressreports/2012countries/ce_BF_Narrative_Report[1].pdf.

[3] Burkina Faso: Epidemiological fact sheets on HIV/AIDS and sexually transmitted disease; 12 2006 WHO Available: http://www.who.int/GlobalAtlas/predefinedReports/EFS2006/EFS_PDFs/EFS2006_BF.pdf.

[4] IeDea, Collaborations ARTC, Avila D, Althoff KN, Mugglin C, Wools-Kaloustian K, Koller M, Dabis F et al Immunodeficiency at the start of combination antiretroviral therapy in low-, middle-, and high-income countries. J Acquir Immune Defic Syndr. 2014;65: e8–16. 10.1097/QAI.0b013e3182a39979 24419071 PMC3894575

[5] World Health Organization. Consolidated guidelines on the use of antiretroviral drugs for treating and preventing HIV infection [Internet] [cited 2013 Aug 16]. Available: http://www.who.int/hiv/pub/guidelines/arv2013/en/. 24716260

[6] World Health Organization. Antiretroviral therapy for HIV infection in adults and adolescents: recommendations for a public health approach Geneva: World Health Organization; 2010. 2010 revision. 23741771

[7] Hoffmann CJ, Schomaker M, Fox MP, Mutevedzi P, Giddy J, Prozesky H, et al CD4 count slope and mortality in HIV-infected patients on antiretroviral therapy: multicohort analysis from South Africa. J Acquir Immune Defic Syndr. 2013;63: 34–41. 10.1097/QAI.0b013e318287c1fe 23344547 PMC3655761

[8] Kiertiburanakul S, Boettiger D, Lee MP, Omar SF, Tanuma J, Ng OT, et al Trends of CD4 cell count levels at the initiation of antiretroviral therapy over time and factors associated with late initiation of antiretroviral therapy among Asian HIV-positive patients. J Intern AIDS Soc. 2014;17: 18804 10.7448/IAS.17.1.18804 24598459 PMC3944639

[9] Venkataramani AS, Thirumurthy H, Haberer JE, Ii YB, Siedner MJ, Kembabazi A, et al CD4+ cell count at antiretroviral therapy initiation and economic restoration in rural Uganda. AIDS. 2014;28: 1221–1226. 10.1097/QAD.0000000000000188 24406678 PMC4092056

[10] Nash D, Wu Y, Elul B, Hoos D, El Sadr W; International Center for AIDS Care and Treatment Programs. Program-level and contextual-level determinants of low-median CD4+ cell count in cohorts of persons initiating ART in eight sub-Saharan African countries. AIDS. 2011;25: 1523–1533. 10.1097/QAD.0b013e32834811b2 21750418 PMC3422866

[11] Fontaine C, Hema A, Kamboule E. L’hôpital de jour du CHU de Bobo Dioulasso: une structure de référence pour la prise en charge des patients infectés par le VIH au Burkina Faso. Med Mal Infect. 2009;40: 393–397. 10.1016/j.medmal.2009.10.018 19951831

[12] Akileswaran C, Lurie MN, Flanigan TP, Mayer KH. Lessons learned from use of highly active antiretroviral therapy in Africa. Clin Infect Dis. 2005;4: 376–385. 10.1086/431482 16007536

[13] ART-LINC Collaboration of International Databases to Evaluate AIDS (IeDEA)^1^, Keiser O, Anastos K, Schechter M, Balestre E, Myer L, Boulle A et al Antiretroviral therapy in resource-limited settings 1996 to 2006: patient characteristics, treatment regimens and monitoring in sub-Saharan Africa, Asia and Latin America. Trop Med Int Health. 2008;13: 870–879. 10.1111/j.1365-3156.2008.02078.x 18373510 PMC3722496

[14] Luo C, Akwara P, Ngongo N, Doughty P, Gass R, Ekpini R, et al Global progress in PMTCT and paediatric HIV care and treatment in low- and middle-income countries in 2004–2005. Reprod Health Matters. 2007;15: 179–189. 17938083 10.1016/S0968-8080(07)30327-3

[15] Mugglin C, Estill J, Wandeler G, Bender N, Egger M, Gsponer T, et al Loss to programme between HIV diagnosis and initiation of antiretroviral therapy in sub-Saharan Africa: systematic review and meta-analysis. Trop Med Int Health. 2012;17: 1509–1520. 10.1111/j.1365-3156.2012.03089.x 22994151 PMC3895621

[16] Staveteig S, Shanxiao Wang, Sara K. Demographic patterns of HIV testing uptake in sub-Saharan Africa. Calverton, MD, ICF International, USA, 2013 (DHS Comparative Reports No. 30). Available: http://dhsprogram.com/pubs/pdf/CR30/CR30.pdf.

[17] Van Rooyen H, Barnabas RV, Baeten JM. High HIV testing uptake and linkage of care in a novel program of home-based HIV counselling and testing with facilitated referral in KwaZulu-Natal, South Africa. J Acquir Immune Defic Syndr. 2013;64: e-1–8. 23714740 10.1097/QAI.0b013e31829b567d PMC3744613

[18] Basset I, Regan S, Luthuli P. Linkage to care following community-based mobile HIV testing compared with clinic-based testing in Umlazi, Durban, South Africa. HIV Med. 2014;15: 367–372. 10.1111/hiv.12115 24251725 PMC4026348

[19] Ortego C, Huedo-Medina TB, Santos P. Sex differences in adherence to highly active antiretroviral therapy: a meta-analysis. AIDS Care. 2012;24: 1519–1534. 10.1080/09540121.2012.672722 22533692

[20] Le VIH/SIDA en Afrique subsaharienne: le point sur l’épidémie et les progrès du secteur de la santé vers l’accès universel. Rapport de situation 2011 OMS, ONUSIDA, UNICEF. Available: http://www.who.int/hiv/pub/progress_report2011/africa/fr/.

[21] Mugglin C, Estill J, Wandeler G, Bender N, Egger M, Gsponer T et al for le DEA Southern Africa. Loss to programme between HIV diagnosis and initiation of antiretroviral therapy in sub-Saharan Africa: systematic review and meta-analysis. Trop Med Int Health. 2012;17: 1509–1520. 10.1111/j.1365-3156.2012.03089.x 22994151 PMC3895621

[22] Kranzer K, Govindasamy D, Ford N. Quantifying and addressing losses along the continuum of care for people living with HIV infection in sub-Saharan Africa: a systematic review. J Int AIDS Soc. 2012;15: 17383 10.7448/IAS.15.2.17383 23199799 PMC3503237

[23] Rosen S, Fox M, Gill CJ. Patient retention in antiretroviral therapy programs in sub-Saharan Africa: a systematic review. PLOS Medicine. 2007;4: 1691–1701. 10.1371/journal.pmed.0040298 PMC2020494 17941716

[24] Sieleunou I, Souleymanou M, Schönenberger AM, Menten J, Boelaert M. Determinants of survival in AIDS patients on antiretroviral therapy in a rural centre in the Far-North Province, Cameroon. Trop Med Int Health. 2009;14: 36–43. 10.1111/j.1365-3156.2008.02146.x 19017309

[25] Kranzer K, Zeinecker J, Ginsberg P. Linkage to HIV care antiretroviral therapy in Cape Town, South Africa. PloS ONE. 2010;5: e13801 10.1371/journal.pone.0013801 21072191 PMC2970551

[26] Wood E, Braitstein P, Montaner JS. Extent to which low-level use of antiretroviral tretment could curb the AIDS epidemic in sub-Saharan Africa. Lancet. 2000;355: 2095–2100. 10902622 10.1016/S0140-6736(00)02375-8

[27] Cohen MS, Chen YQ, McCauley M. Prevention of HIV-1 infection with early antiretroviral therapy. New Engl J Med. 2011:365: 493–505. 10.1056/NEJMoa1105243 21767103 PMC3200068

[28] OMS, ONUSIDA, UNICEF. Le point 2013 OMS sur le traitement de l’infection à VIH dans le monde: résultats, impact et opportunités, June 2013. Available: http://www.who.int/hiv/pub/progressreports/update2013/fr/.

[29] Granich RM, Gilks CF, Dye C. Universal voluntary HIV testing with immediate antiretroviral therapy as a strategy for elimination of HIV transmission: a mathematical model. Lancet. 2009;373: 48–57. 10.1016/S0140-6736(08)61697-9 19038438

[30] Kato M, Granich R, Bui DD. The potential impact of expanding antiretroviral therapy and combination prevention in Vietnam: towards elimination of HIV transmission. J Acquir Immune Defic Syndr. 2013;63: e142–149. 10.1097/QAI.0b013e31829b535b 23714739 PMC3814627

[31] Fatti G, Grimwwod A, Bock P. Better antiretroviral therapy outcomes at primary healthcare facilities: an evaluation of three tiers of ART services in four South African provinces. Plos One. 2010;5: e12888 10.1371/journal.pone.0012888 20877631 PMC2943483

[32] Direction générale du Trésor website. Available: https://www.tresor.economie.gouv.fr/4982_situation-economique-du-burkina-faso. Accessed 2012 February 27.

[33] De Beaudrap P, Thiam M, Diouf A. Risk of virological failure and drug resistance during first and second-line antiretroviral therapy in a 10-year cohort in Senegal: results from the ANRS 1215 cohort. J Acquir Immune Defic Syndr. 2013;62: 381–387. 10.1097/QAI.0b013e31827a2a7a 23117504

[34] WHO HIV drug resistance report 2012. Genève, Organisation mondiale de la santé, 2012. Available: http://apps.who.int/iris/bitstream/10665/75183/1/9789241503938_eng.pdf

[35] Essai ANRS 12 169, 2-LADY. Evaluation de trois stratégies de traitement antirétroviral de 2ème ligne en Afrique (Cameroun, Sénégal, Burkina Faso). Available: http://www.anrs.fr/content/download/2662/15627/file/ANRS%2012169%202-LADY.pdf.

[36] Essai ANRS 12 269, THILAO. Renforcement de l’observance et traitement à base de darunavir et raltegravir chez des adultes infectés par le VIH-1 en échec virologique de deuxième ligne de traitement antirétroviral en Afrique sub-Saharienne: Cohorte thérapeutique THILAO (« Third Line Antiretroviral Optimization »), (Burkina Faso, Mali, Sénégal, Côte d’Ivoire, Cameroun). Available: www.anrs.fr/content/download/4104/21815/file/1-4-Eholie_THILAO.pdf.

[37] OMS, résumé des principales caractéristiques et recommandations, juin 2013: Lignes directives unifiées sur l’uitilisaion des antirétroviraux pour le traitement et la prévention de l’infection à VIH. Available: http://www.who.int/hiv/pub/guidelines/arv2013/short_summary/fr/.

[38] Ivers LC, Cullen KA, Freedberg KA. HIV/AIDS, undernutrition, and food insecurity. Clin Infect Dis. 2009;49: 1096–1102. 10.1086/605573 19725790 PMC2831619

[39] Etard JF, Laniece I, Fall MB, Cilote V, Blazejewski L, Diop K, et al A 84 month follow-up of adherence to HAART in a cohort of adult Senegalese patients. Trop Med Int Health. 2007;12: 1191–1198. 17956501 10.1111/j.1365-3156.2007.01910.x

[40] Bärnighausen T, Chaiyachati K, Chimbindi N. Interventions to increase antiretroviral adherence in sub-Saharan Africa: a systematic review of evaluation studies. Lancet Infect Dis. 2011;11: 942–951. 10.1016/S1473-3099(11)70181-5 22030332 PMC4250825

[41] Elul B, Basinga P, Nuwagaba-Biribonwoha H. High level of adherence and viral suppression in a nationally representative sample of HIV-infected adults on antiretroviral therapy for 6, 12 and 18 months in Rwanda. PloS One. 2013;8: e53586 10.1371/journal.pone.0053586 23326462 PMC3541229

